# *Candida glabrata*: new tools and technologies—expanding the toolkit

**DOI:** 10.1093/femsyr/fov066

**Published:** 2015-07-23

**Authors:** Hsueh-lui Ho, Ken Haynes

**Affiliations:** Biosciences, University of Exeter, Stocker Road, Exeter, Devon EX4 4QD, UK

**Keywords:** *Candida glabrata*, virulence, tools, technologies, *Candida*, *S. cerevisiae*

## Abstract

In recent years, there has been a noticeable rise in fungal infections related to non-*albicans Candida* species, including *Candida glabrata* which has both intrinsic resistance to and commonly acquired resistance to azole antifungals. Phylogenetically, *C. glabrata* is more closely related to the mostly non-pathogenic model organism *Saccharomyces cerevisiae* than to other *Candida* species. Despite *C. glabrata*'s designation as a pathogen by Wickham in 1957, relatively little is known about its mechanism of virulence. Over the past few years, technology to analyse the molecular basis of infection has developed rapidly, and here we briefly review the major advances in tools and technologies available to explore and investigate the virulence of *C. glabrata* that have occurred over the past decade.

## Abstract (precis)

New tools and technologies to investigate the virulence of *Candida glabrata*.

## Graphical abstract

**Graphical Abstract Figure.**
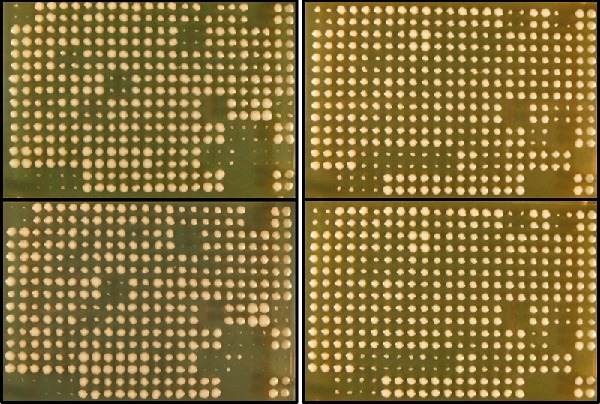
New tools and technologies to investigate the virulence of *Candida glabrata*.

## INTRODUCTION TO *CANDIDA* SPECIES: A SIGNIFICANT HEALTHCARE PROBLEM

Modern technology has helped advance our life expectancy greatly, but this has been accompanied by an increased incidence of fungal infections, which can at least be partially attributed to an increase in both the aged population and the number of immune-compromised individuals. Studies suggest that systemic fungal infections cost the healthcare industry approximately $2.6 billion per year in the USA alone (Wilson *et al.* 2002). The most frequently encountered systemic fungal infections are those caused by the filamentous fungus *Aspergillus* species and the yeasts, *Cryptococcus* and *Candida* species (Pelz *et al.* 2000; Wilson *et al.* 2002; Pfaller and Diekema 2007; Pfaller *et al.* 2010; Diekema *et al.* 2012). *Candida* species are the fourth most common cause of bloodstream infections in the USA (Morgan *et al.* 2005; Pfaller and Diekema 2007), and of the $2.6 billion spent on treating systemic fungal infections, the total costs are highest for candidosis, at a staggering $1.7 billion (Wilson *et al.* 2002). In the USA, candidosis accounts for approximately 75% of all systemic fungal infections and is associated with a crude mortality rate of 46–75% (Pelz *et al.* 2000; Wilson *et al.* 2002). Although *Candida albicans* is the most common aetiological agent, the past two decades have shown a rise in the incidence of non-*albicans Candida* species (Pelz *et al.* 2000; Wilson *et al.* 2002; Pfaller and Diekema 2007; Pfaller *et al.* 2010; Diekema *et al.* 2012). Of interest, in the rise of non-*albicans Candida* species, is *C. glabrata* which has been shown to be associated with longer hospital stays and higher costs than *C. albicans* (Moran *et al.* 2010). This could partially be attributed to *C. glabrata*'s intrinsic and acquired resistance to commonly used azole antifungals (Fidel, Vazquez and Sobel 1999; Pfaller and Diekema 2007; Pfaller *et al.* 2010; Diekema *et al.* 2012). Worryingly, *C. glabrata* isolates exhibiting a decreased susceptibility to echinocandins (considered the first line treatment for *C. glabrata* infections; Pappas *et al.* 2009) and Amphotericin B, considered the gold standard treatment for fungal infections, have also been reported (Krogh-Madsen *et al.* 2006; Pfaller *et al.* 2012; Alexander *et al.* 2013). This demands that we understand the molecular mechanisms underpinning this rise in incidence as a necessary precursor to developing novel durable therapies for infections caused by *C. glabrata*.

**Figure 1.**
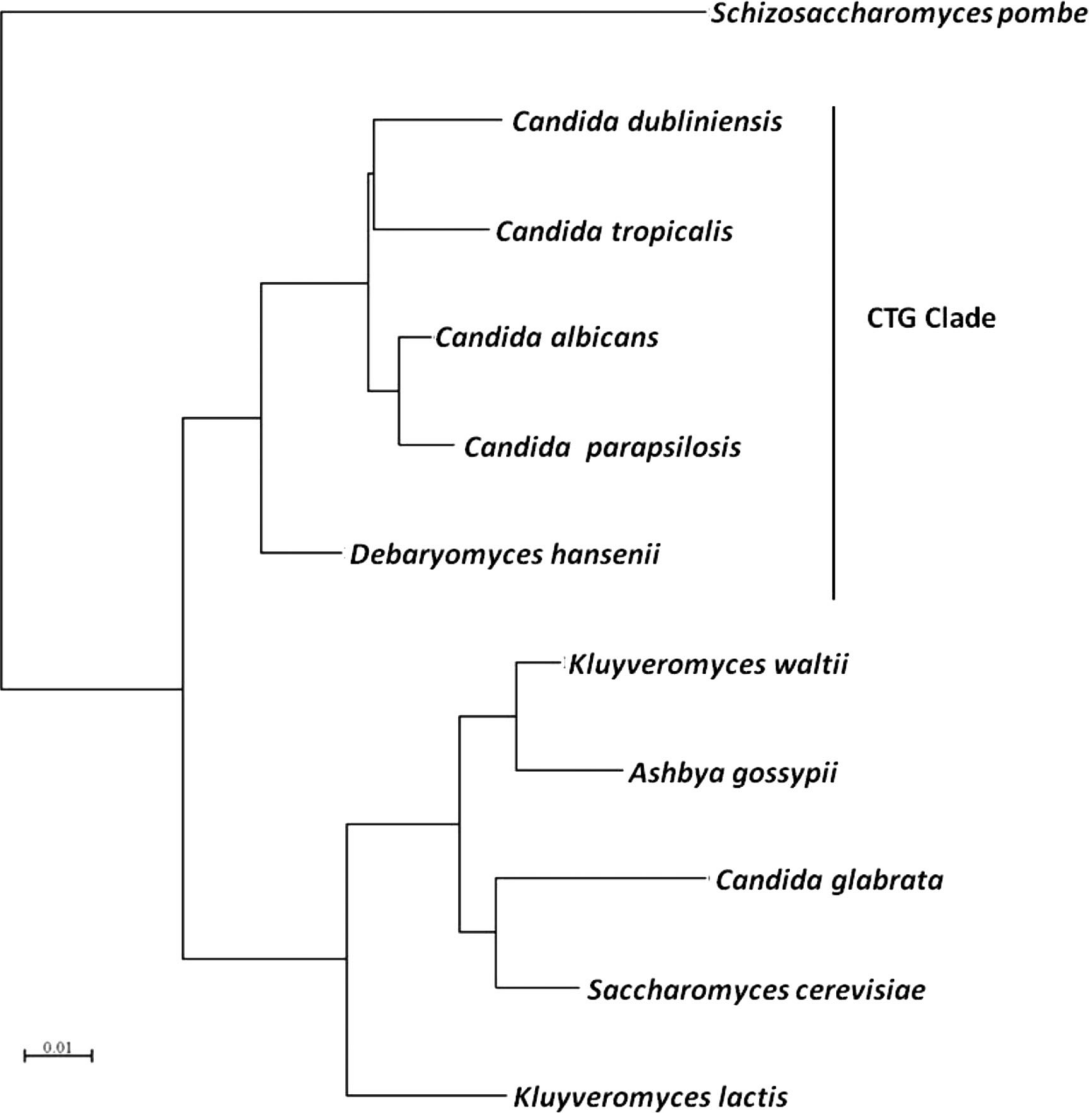
18S phylogeny of five *Candida* species and six hemiascomycetes. 18S sequences and phylogram were aligned and generated using Seaview version 4.5.3 (Gouy, Guindon and Gascuel 2010). *Candida glabrata* is phylogentically most closely related to *S. cerevisiae* and is not a member of the CTG clade which other *Candida* species belong to.

*Candida glabrata* is a commensal of the oral cavity and human gut forming part of the normal microflora of healthy individuals (Fidel, Vazquez and Sobel 1999; Ahmad *et al.* 2014). Phylogenetically, it is more closely related to *Saccharomyces cerevisiae* than to any other *Candida* species, see Fig. 1, and unlike most well-studied *Candida* species, it belongs to the non-CTG clade species (Dujon *et al.* 2004; Ahmad *et al.* 2014). *Candida glabrata* is strictly haploid and grows as a unicellular yeast, and in contrast to *C*. *albicans* does not form pseudo hyphae at 37°C *in vivo* or under normal growth conditions *in vitro* (Fidel, Vazquez and Sobel 1999). However, *C. glabrata* has been reported to form pseudo hyphae growth when grown under nitrogen starvation at both 30 and 37°C *in vitro* on solid media (Csank and Haynes 2000; Calcagno *et al.* 2003). Furthermore, *C. glabrata* has no known documented sexual cycle although both mating types are commonly found and its genome contains homologues of the majority of the genes involved in mating in *S. cerevisiae* (Muller *et al.* 2008). In contrast to the aggressive strategies used by other fungal pathogens, such as *C. albicans*, *C. glabrata* uses a combination of immune evasion and persistence to invade and colonize its host; for example, it evades the host immune system by allowing itself to be taken up by macrophages where it can continue to proliferate (Kaur, Ma and Cormack 2007; Roetzer *et al.* 2010; Seider *et al.* 2011; Brunke and Hube 2013) and only induces transient proinflammatory cytokine responses (Jacobsen *et al.* 2010). Despite these insights, the mechanism of virulence at the molecular level is still not well understood in *C. glabrata* making it difficult to identify candidate proteins as potential drug targets for effective treatment of infections caused by this yeast (Silva *et al.* 2011; Ahmad *et al.* 2014).

Compared with the model organism *S. cerevisiae, C. glabrata* has been less well studied, and as such there are fewer molecular tools and resources available to interrogate its biology; see Table 1 for a summary. Research into *C. glabrata* has been hampered by the lack of a known sexual cycle, preventing classical genetic analysis, which has been used widely in many fungal species such as *S. cerevisiae* and *Aspergillus* species to dissect genes and pathways (Muller *et al.* 2008). Similarly, the power of the synthetic genetic array (SGA) analysis, which has revolutionized research by enabling genetic interactions networks to be mapped, cannot be used in *C. glabrata* as the method itself exploits the presence of a sexual cycle (Tong *et al.* 2001; Costanzo *et al.* 2010). Furthermore, genetic work involving generation of deletion/tagged strains in *C. glabrata* has been hampered by its preference for non-homologous end joining (NHEJ) over homologous recombination, which is required for targeted integration of a DNA fragment at double-strand breaks (DSB), thus making gene targeting much less efficient than in *S. cerevisiae* (Ueno *et al.* 2007; Corrigan *et al.* 2013; Cen, Fiori and Dijck 2015). In the absence of these approaches, much work has exploited knowledge from *S. cerevisiae* as a basis for formulating hypotheses and then extrapolating back to *C. glabrata*. For example, the majority of the annotated genes in *C. glabrata* have not been experimentally annotated, rather their encoded functions have been predicted based on homology to their *S. cerevisiae* orthologues (Dujon *et al.* 2004). Those that have been experimentally annotated for *C. glabrata*, such as *MNN2, MNN11, MSN*2/4, *SHO1* or *PBS*2, have often been done so by complementing the *C. glabrata* orthologue into the *S. cerevisiae* knockout and vice versa to confirm encoded functions (Gregori *et al.* 2007; Roetzer *et al.* 2008; West *et al.* 2013). However, caution should be taken from such approaches as the function of a protein can vary from species to species; for example, *C. glabrata* Hog1, although having a very similar function to its *S. cerevisiae* orthologue, has also been found to modulate resistance to weak organic acids in *C. glabrata* (Gregori *et al.* 2007). Furthermore, MacCallum *et al.* (2006) showed that despite a deletion of *ACE2* in *S. cerevisiae, C. albicans* and *C. glabrata* having a similar phenotype *in vitro*, when examined in a murine model of disseminated infection, *C. glabrata ace2* was found to be hypervirulent while *C. albicans ace2/ace2* homozygous null mutants were slightly attenuated. Even more recently, Varshney *et al.* (2015) discovered that while Sch9 was important for chromosome segregation in *C. albicans*, its homologue in *S. cerevisiae* plays no apparent role in chromosome segregation despite their sequence similarity. These observations strengthen the case for investigating genes of interest in the species of interest as well as in model organisms.

**Table 1.** Summary of tools and resources available for investigating *S. cerevisiae* and *C. glabrata*.

Tools and resources	Available in *S. cerevisiae*	Available in *C. glabrata*	Reference
Sequenced and annotated genome	✓	✓	Skrzypek and Hirschman (2011); Inglis *et al.* (2012)
Tet-regulatable library of essential genes	✓	×	Mnaimneh *et al.* (2004)
Knockout library of non-essential genes	✓	Partial collection available (2015)	Giaever *et al.* (2002); Winzeler *et al.* (1999)
Green fluorescent protein (GFP) tagged library	✓	×	Huh *et al.* (2003)
HA- tagged library	✓	×	Kumar *et al.* (2002)
Tap-fusion library	✓	×	Ghaemmaghami *et al.* (2003)
GST-tagged library	✓	×	Sopko *et al.* (2006)
YFP- fusion kinase collection	✓	×	Ma *et al.* (2008)
Gateway ORFeome	✓	×	Gelperin *et al.* (2005)
Proteome interactome collection	✓	×	Tarassov *et al.* (2008)
Yeast cross and capture system collection	✓	×	Suter *et al.* (2007)
Insertional mutant collection	✓	Partial collection available (approx. 25% of the genome)	Ross-Macdonald *et al.* (1999); Castano *et al.* (2003)
Synthetic histone collection	✓	×	Dai *et al.* (2008)
DAmP collection	✓	×	Breslow *et al.* (2008)
RNA-seq data	✓	✓	Aoyama *et al.* (2014); Linde *et al.* (2015); Nookaew *et al.* (2012)
Yeast -2- hybrid data	✓	×	Ito *et al.* (2001)
Genetic interaction data	✓	×	Costanzo *et al.* (2010)
CRISPR compatible plasmids	✓	×	Bao *et al.* (2014); Zalatan *et al.* (2014)
RNAi compatible plasmids	✓	×	Crook, Schmitz and Alper (2014); Drinnenberg *et al.* (2009)
Gateway compatible destination plasmids	✓	Limited Gateway Destination vectors available –so far for complementation only (2015)	Flagfeldt *et al.* (2009); Schwarzmüller *et al.* (2014); Thorne *et al.* (2011)
Plasmid collection for constructing gene knockouts/ fusions (e.g. GFP integration)	✓	✓	Janke *et al.* (2004); Schwarzmüller *et al.* (2014)
Generation of the synthetic yeast genome	✓	×	Annaluru *et al.* (2014)

### The *C. glabrata* knockout collection

Deletion libraries are a useful tool to investigate gene function. Since the release of the *S. cerevisiae* Yeast Knockout Collections (YKO), which comprises 5916 individual genes knocked out in one or more of four backgrounds (*MAT***a**, *MAT*α, heterozygous diploid, and homozygous diploid) (Winzeler *et al.* 1999; Giaever *et al.* 2002), many high-throughput screens have been undertaken using these libraries producing a wealth of information that has led to functions being assigned to previously unannotated proteins. For example, Giaever *et al.* (2002) identified the previously uncharacterized gene *YJL200C*, now termed *ACO2*, to encode the enzyme most likely to be involved in the second step of the lysine biosynthetic pathway (the conversion of homocitrate to homo-*cis*-iconitate) when they pooled the YKO library and grew it in media lacking threonine, tryptophan or lysine. Costanzo *et al.* (2010) used the YKO library in SGA analysis to create a map of the genetic interactions in *S. cerevisiae* before interrogating connectivity within the map to predict the function encoded by uncharacterized genes. Taking this approach, they successfully predicted that the products of *PAR32, ECM30* and *UBP15* are involved in Gap1 sorting. While Kemmeren *et al.* (2005) have developed a bioinformatic tool to predict gene function by integrating 125 high-throughput data sets, including gene-deletion phenotype data generated from screening the YKO collection, into one database and mined the data for multiple pairwise relationships under a variety of different criteria. Using this technique, they generated 543 predictions for genes encoding proteins with unknown functions and experimentally confirmed a selection. For instance, they confirmed their prediction that the uncharacterized gene *YGR205W* is involved in stress response as a *ygr205w* null resulted in increased thermotolerance (Kemmeren *et al.* 2005). These types of screens and analyses can easily be extended to any organism in which a deletion collection has been constructed. Indeed, the deletion collection for *Schizosaccharomyces pombe*, reported by Kim *et al.* shortly after the *S. cerevisiae* deletion collection**, has been used in genome-wide analyses producing a wealth of novel information including the identification of a new set of genes encoding functions involved in transcription and translation in *S. pombe* (Kim *et al.* 2010).

Gene targeting in *C. glabrata* has been hindered by its high preference for NHEJ at DSB over homologous recombination which is required for target integration of DNA fragments (Ueno *et al.* 2007; Corrigan *et al.* 2013). Although attempts have been made to overcome this problem in *C. glabrata*, for example, Ueno *et al.* (2007) generated a reversible *YKU80* disruption strain (Yku80 is involved in the NHEJ pathway) thereby increasing gene targeting efficiency by 5.1% using 40-bp flanking homologous DNA, this strain has not gained widespread use from the community. This is in contrast to other species where knockout *KU70/ KU80* strains (key genes involved in NHEJ in eukaryotes) are frequently utilized to generate knockouts, for example, *Magnaporthe grisea, Zymoseptoria tritici* and *Aspergillus fumigatus* (da Silva Ferreira *et al.* 2006; Villalba *et al.* 2008; Kershaw and Talbot 2009; Sidhu *et al.* 2015). One likely reason that the *C. glabrata* community has not widely used the reversible *YKU80* disruption strain for generating knockouts is because the target efficiency is only increased by 5.1% and disruption of *YKU80* leads to synthetic sickness or lethality in *S. cerevisiae* (Ueno *et al.* 2007); furthermore, deletions of *YKU70/80* in *C. glabrata* have been shown to affect subtelomeric silencing (Rosas-Hernández *et al.* 2008; Cen, Fiori and Dijck 2015). In contrast, a deletion of *KU80/ 70* in *M. grisea* leads to an increase in gene targeting efficiency from 5 to 80% and the nulls display wild-type phenotypes with regard to pathogenicity, growth, sporulation and mating (Villalba *et al.* 2008; Kershaw and Talbot 2009). More recently, Cen Fiori and Dijck (2015) have constructed a *lig4* mutant (Lig4 is a DNA ligase involved in NHEJ) and shown that gene targeting efficiency increased up to 35 times compared to the parental strain when using 40-bp flanking homologous DNA fragments. Furthermore, they noted that a deletion of *LIG4* posed no deleterious effect on the strain compared to the parental when grown under a variety of different conditions including cell wall stress, antifungals and DNA damage (Cen, Fiori and Dijck 2015). In addition, to avoid any phenotypes caused by the absence of *LIG4*, Cen Fiori and Dijck (2015) constructed a reintegration cassette that can be used to reintegrate *LIG4* at its original locus. As gene targeting can be performed more efficiently in this newly constructed *lig4* strain compared to the *yku70/80* strains and does not appear to cause any phenotype upon its deletion, it will be interesting to see if the *Candida* community will adopt this strain for use in high-throughput knockout generation in the near future.

Until recently, no deletion collection existed for *C. glabrata*; thus, research has focused on C. *glabrata* genes known to impact virulence in other pathogens, or on orthologues of *S. cerevisiae* genes that may or may not encode functions important for infection. For example, de Groot *et al.* (2008) examined the cell wall of *C. glabrata* for novel adhesin-like cell wall proteins based on the important role the cell wall plays for survival in different environmental conditions and because a number of fungal cell wall proteins have been shown to be instrumental in adhesion to human tissues, such as the Als proteins, Eap1 and Hwp1 in *C. albicans*. While this approach has revealed some aspects of *C. glabrata* biology, many genes in this species remain uncharacterized, especially those that are specific to *C. glabrata*; these genes may be vital for *C. glabrata*'s pathogenicity and ability to survive within the host. Another approach researchers currently employ to investigate virulence in *C. glabrata* and that takes into account of genes that may be specific to *C. glabrata* is to construct pooled libraries of randomly generated mutant strains and then to screen the library for a specific phenotype. Indeed, this approach was used by Cormack, Ghori and Falkow (1999) when they discovered the main adhesin, Epa1, in the Epa family of adhesins for *C. glabrata*, while generating a random *C. glabrata* library of insertional mutants and screening them for their ability to adhere to HEp2 cells. Since then, more than 20 genes have been found to belong to the Epa family of adhesins in *C. glabrata* and some of these genes have been found to encode functional adhesins (Cormack, Ghori and Falkow 1999; De Las Peñas *et al.* 2003; Castaño *et al.* 2005). A disadvantage in using this approach is that a specific phenotype believed to be important for virulence is investigated; thus, those phenotypes that may not obviously be deemed to be important for virulence are not examined. In addition, these types of approaches are more time consuming as they require the mutation/deletion/insertion of interest to be identified after isolation via sequencing for example, whereas using a known deletion collection/ library omits this step.

In 2014, Schwarzmüller *et al.* (2014) published the first large-scale phenotypic screen of a *C. glabrata* deletion collection revealing several novel antifungal tolerance genes. The *C. glabrata* deletion collection currently comprises 619 strains representing approximately 12% of the predicted genome (Schwarzmüller *et al.* 2014). Each knockout strain contains molecular barcodes to enable pooling and/or competition experiments. A recyclable *NAT1* marker was utilized to generate each knockout allowing the *NAT1* marker to be used for further deletions in the same strain. The majority of the knockouts were constructed in *C. glabrata* HTL, a *his3, leu2 trp1* auxotrophic derivative of the type strain *C. glabrata* ATCC 2001. However, approximately 195 transcription factor mutants were made in a single *his3* auxotrophic background (Schwarzmüller *et al.* 2014). Pertinently, Jacobsen *et al.* have shown that loss of *HIS3, LEU2, TRP1* or a combination of all three in *C. glabrata* does not impact its virulence**, unlike deletion of the *URA3* marker in *C. albicans* which is known to affect its virulence (Lay *et al.* 1998; Brand *et al.* 2004; Jacobsen *et al.* 2010). Thus, these strains can be used to analyse the virulence of *C. glabrata*. Indeed, Brunke *et al.* (2015) recently took 416 strains from the *C. glabrata* deletion collection and examined them for virulence using an immunodeficient *Drosophila melanogaster* model and discovered that an intact cell wall was important for *C. glabrata* virulence as many genes involved in the maintenance of cell wall integrity were amongst the most strongly attenuated mutants in their screen. An additional strength of this deletion collection is the optimized gene disruption protocol which was developed for strain construction, and which will facilitate additions to the library.

Evidence of the usefulness of this deletion collection can already be seen in recent published work by Schwarzmüller *et al.* (2014), who described large-scale phenotypic screens that identified novel genes encoding functions implicated in azole tolerance, including *YPK1* and *KTR2* where the null strains showed hypersensitivity to azoles, and which have not previously been associated with antifungal tolerance. Furthermore, they identified 28 knockout strains that are hypersensitive to caspofungin and have not been described in *S. cerevisiae* nor *C. albicans*, implying that *C. glabrata* may have unknown mechanisms of caspofungin resistance (Schwarzmüller *et al.* 2014). In addition, Kasper *et al.* (2014) utilized the deletion collection to identify genes required for environmental alkalinization and showed that protein mannosylation may play a key role in *C. glabrata's* ability to alter the phagosomal environment allowing it to survive and proliferate intracellularly. Considering most *C. glabrata* research is currently based on data from *S. cerevisiae* and a small number of fungal pathogens, it is likely that these genes would have taken many years to come to light without the aid of a deletion collection. Although the *C. glabrata* deletion collection currently comprises only 12% of the genome, advances have already been made in understanding the virulence mechanisms of *C. glabrata* and additional strains have been constructed (K. Haynes *et al.*, unpublished). It is hoped that the community will add to this collection in the future by utilizing the optimized gene disruption protocol and the background strains to deliver a more complete deletion collection for future high-throughput assays and thus facilitate a greater understanding of *C. glabrata*'s successful pathogenic strategies.

### A plasmid toolbox for *C. glabrata*

**Figure 2.**
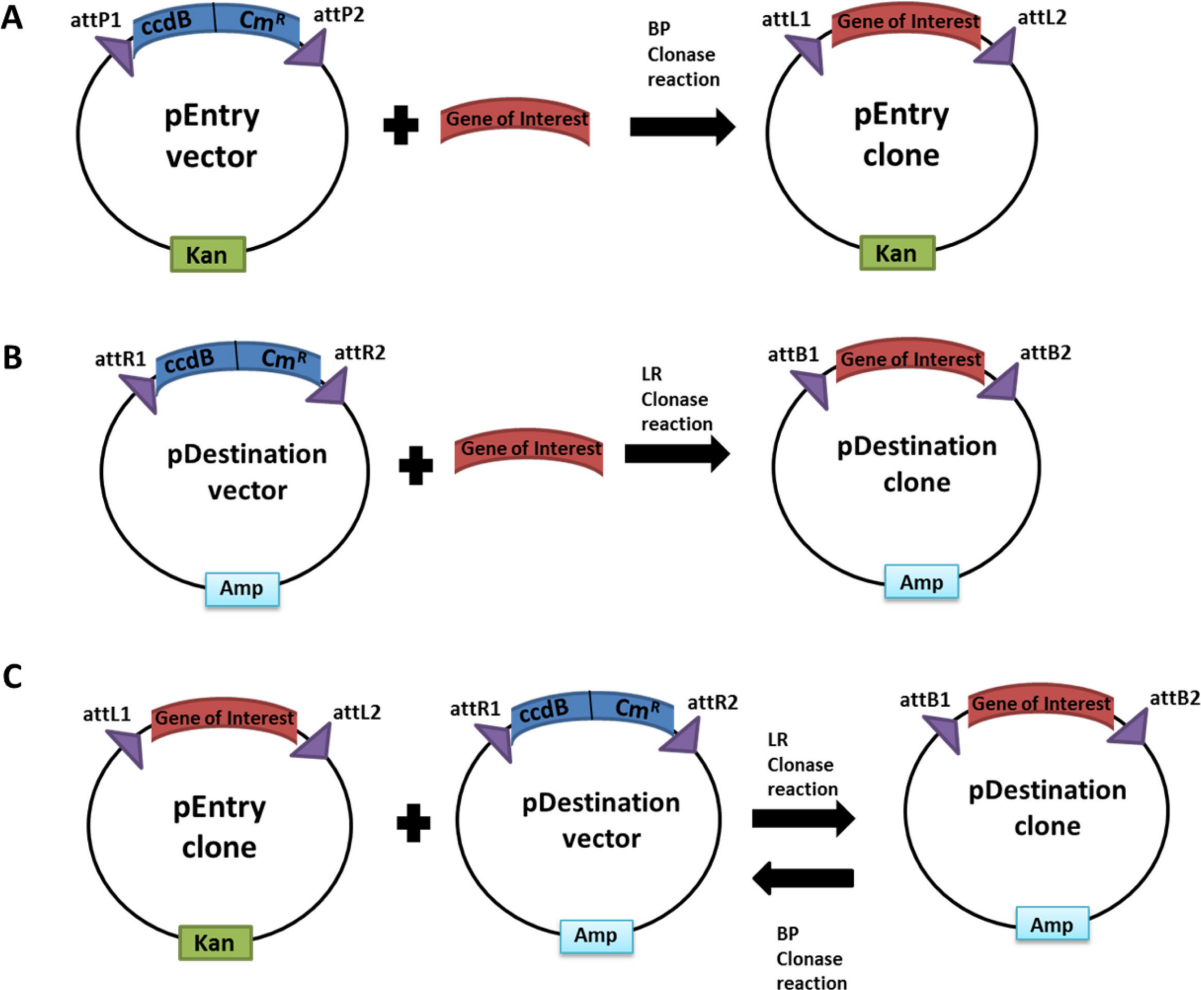
Overview of the Gateway system. The Gateway system facilitates high-efficiency transfer of genes between different Gateway vectors via site-specific recombination. (**A**) The gene of interest is cloned in between the attP1/2 sites on the pEntry vector via a BP Clonase reaction to produce a pEntry clone. The pEntry vector harbours a bacterial ‘death’ gene (ccdB) that is exchanged for the gene of interest during the generation of the pEntry clone and transformation of *E.coli* that are sensitive to the ccdB effects allows for selection of the pEntry clones. (**B**) The gene of interest can also be cloned directly between the attR1/2 sites of a pDestination vector (containing features of interest such as protein tags, etc.) via a LR Clonase reaction to generate an expression vector thereby omitting the initial step of generating a pEntry clone. The pDestination vectors also contain the bacterial ‘death’ gene, ccdB, to enable selection of the pDestination clones. (**C**) The generated pEntry clone can be mixed with any available pDestination vector via a LR Clonase reaction to generate pDestination clones. This reaction is reversible using the BP Clonase reaction. pDestination clones with the gene of interest can be used to regenerate/generate the pEntry clones.

Plasmids have formed an essential part of the molecular biology toolkit since Cohen *et al.* (1973) reported that individual genes can be cloned and isolated by enzymatically fragmenting DNA molecules and ligating them into autonomously replicating circular genetic elements before introducing them into bacteria. Unsurprisingly, the model organism, *S. cerevisiae*, has one of the most diverse plasmid toolkits available for ease of investigating and manipulating genes, from expression plasmids to reporter plasmids. For example, Janke *et al.* (2004) released a plasmid suite for PCR-based tagging of *S. cerevisiae* genes that consisted of new fluorescent proteins (a yeast optimized version of the red fluorescent protein, named RedStar2), additional positive selection markers (*hphNtI, natNT2*) and nine promoter substitution cassettes. This toolkit has recently been expanded with the introduction of the Gateway® Technology which facilitates high-efficiency transfer of genes between different Gateway vectors by site-specific recombination (Hartley 2000). A drawback to the system is that it requires the initial insertion of the gene of interest into a plasmid with the two flanking Gateway recombination sites (*att*L1 and *att*L2) to produce an initial Gateway Entry clone (Hartley 2000). However, once an Entry clone is available, it is possible to shuttle the gene of interest to any available Gateway adapted vector; furthermore, this process is bidirectional (Hartley 2000), see Fig. 2 for an overview of the Gateway Technology. Utilizing Gateway Technology, Gelperin *et al.* constructed the MORF (Moveable ORF) library, a library containing 5854 *S. cerevisiae* ORFs as C-terminal His_6_-HA fusions under regulated control in Gateway compatible expression plasmids. Using this collection, they performed the first systematic screen for glycosylated proteins in *S. cerevisiae* and identified 454 new candidate glycoproteins (Gelperin *et al.* 2005). The MORF library was further complemented by a suite of 288 Gateway vectors, released in 2007 by Alberti *et al.*, specifically designed to facilitate the transfer of ORFs from the MORF library into destination vectors designed for a variety of applications, for example protein localization or protein immunoprecipitation (Flagfeldt *et al.* 2009). This new suite of Gateway vectors includes a choice of two promoters (inducible or constitutive) and options for N- or C-terminal fusion to various protein affinity tags (HA, TAP) or fluorescent proteins (EGFP, ECFP, Cerulean or DsRed) as well as high- or low-copy origins of replication (Flagfeldt *et al.* 2009). Furthermore, Gateway ORFeomes and compatible vectors have been created in other species including *D. melanogaster, Caenorhabditis elegans* and *C. albicans* (Reboul *et al.* 2003; Akbari *et al.* 2009; Chauvel *et al.* 2012), enabling protein function to be examined in multiple species and thus to assessment of functional conservation between species.

Although a full *C. glabrata* ORFeome is yet to be published, the Gateway system is already being utilized by *C. glabrata* researchers to investigate genes of interest. Schwarzmüller *et al.* (2014) employed Gateway compatible expression vectors to confirm that the caspofungin sensitivity phenotype observed in 12 of their *C. glabrata* knockout strains was due to the absence of each individual gene, and that the phenotype was restored upon the insertion of an expression plasmid expressing each gene of interest. Similarly, Thorne *et al.* (2011) utilized a Gateway adapted yeast-2 hybrid system to experimentally validate predicted *C. glabrata* and *S. cerevisiae* protein–protein interactions (PPIs) while investigating the utility of evolutionary correlation between presence and absence of genes in a group of species as a method to predict potential PPIs. With the advances in postgenomic tools, large high-throughput data sets are regularly produced; for example, Aoyama *et al.* (2014) used RNA-seq to investigate transcriptional initiation in *C. glabrata* and predicted 50 new *C. glabrata* genes. Yet in the absence of a *C. glabrata* ORF library, any set of genes that emerge from large-scale experiments have to be prioritized for investigation individually as constructs will have to be produced for each gene. The construction of a *C. glabrata* Gateway adapted ORFeome would alleviate this, as any gene of interest could easily be shuttled into a range of different destination vectors thereby accelerating research into multiple genes. Indeed, a partial *C. glabrata* Gateway ORFeome has been used to examine conservation of PPIs between *C. glabrata* and *S. cerevisiae* (Ho and Huvet *et al.*, unpublished) and to perform a pooled overexpression screen to identify novel *C. glabrata* genes important for survival under stress conditions (Ho *et al.*, unpublished).

### Invasive infection models to investigate *C. glabrata* virulence?

The most established invasive infection models for analysis of traits associated with the establishment of candidosis are the intravenous (IV) challenge and gastrointestinal colonization with subsequent dissemination mouse models, reviewed in detail by Szabo and MacCallum (2011). While both mouse models enable host–pathogen interactions to be explored *in vivo* and allow comparison of the ability of mutants to progress disease via measurement of organ fungal burden, they come with caveats. The use of mammalian models requires careful ethical consideration, and has a significant economic cost. For example, Becker *et al.* (2010) assayed the virulence of 177 *C. albicans* strains in a mouse model and identified 102 genes that play a role in host survival and establishment of an infection, including genes that encode known antifungal drug targets such as *FKS1* and *ERG1*, validating the results of the screen for discovering potential novel drug targets. In this study, each *C. albicans* strain examined required 15 mice necessitating the use of a minimum of 2655 mice, excluding controls, to complete the screen (Becker *et al.* 2010). To implement this screen for the partial *C. glabrata* deletion collection would require 9285 mice to assay only 12% of *C. glabrata* genes. This is clearly impractical on a global scale and hence different approaches based on pools of mutants (Hensel *et al.* 1995; Cormack, Ghori and Falkow 1999; Winzeler *et al.* 1999; Chan *et al.* 2000; Giaever *et al.* 2002; Steinmetz *et al.* 2002; Kaur, Castaño and Cormack 2004), and/or alternative virulence models are urgently needed if global analyses of virulence are to be undertaken. Furthermore, as *C. glabrata* seems unable to invade or damage (non-phagocytic) host cells to any measureable extent (Seider *et al.* 2011; Brunke and Hube 2013), there is a lack of good *in vitro* invasion/ damage model systems available to explore *C. glabrata* virulence. Thus, researchers have started examining other hosts, mostly invertebrates, as alternative model systems for analysis of candidosis. Although, an invertebrate host may not appear relevant to human diseases, they possess an innate immune system that shares many similarities with the innate immune response of mammals (Kavanagh and Reeves 2004). For instance, the immune function of Toll, which led to the discovery of toll-like receptors, key molecules that alert the immune system to the presence of microbial infections, was first described in *D. melanogaster* as important to mediating fly immunity to *A. fumigatus* infection, by activating the synthesis of antimicrobial peptides (Lemaitre *et al.* 1996).

The first insect virulence model developed for *Candida* infections was *Galleria mellonella* (wax moth) in which the fungi are injected into the proleg of larvae and survival is monitored over a short time period (Cotter, Doyle and Kavanagh 2000). Subsequent experiments have shown that, with regard to *C. albicans* infections, the *Galleria* model produced results similar to those found in mouse infection models (Cotter, Doyle and Kavanagh 2000; Brennan *et al.* 2002; Fuchs *et al.* 2010a). For example, Brennan *et al.* (2002) infected *G. mellonella* larvae with a *C. albicans cdc35/cdc35* mutant strain and found that all larvae survived 48 hours, compared with 100% mortality in larvae infected with the wild-type parental strain, *C. albicans* SC5314. Similarly, Rocha *et al.* (2001) have previously shown that mice infected with *C. albicans cdc35/cdc35* in the IV model of candidosis had 100% survival 42 days post-infection, while complete mortality was observed in mice infected with a *C. albicans cdc35/cdc35* reconstituted with a plasmid expressing *CDC35* 16 days post-infection. Furthermore, Brennan *et al.* used the *G. mellonella* infection model to distinguish between avirulent, virulent and reduced virulence *C. albicans* strains. They observed that *C. albicans hst7/hst7* was as virulent as the wild type (100% mortality at 48 h), while *cpp1/cpp1* mutants were reduced in virulence compared to the wild type (90% mortality at 48 h) (Brennan *et al.* 2002). Again, this correlates with previous mouse data where 50% of the mice survived 35 days post-infection with *C. albicans cpp1/cpp1* (Csank *et al.* 1997), while 100% mouse mortality was observed 6 days post-infection with *C. albicans hst7/hst7* (Leberer *et al.* 1996). These studies clearly demonstrate that the *G. mellonella* virulence model can be used as a proxy for murine infection caused by *C. albicans*.

The *Galleria* model is attractive for analysis of virulence for a number of reasons: (1) large numbers of larvae can be infected with each mutant strain increasing the statistical power of the assay; (2) the infection experiments are simple to perform and inexpensive; and (3) unlike many other insect hosts, *G. mellonella* can be maintained at a temperature range from 25 to 37°C, so facilitating analysis at or near to human body temperature. This is an important consideration as temperature can affect gene expression and thus the induction/repression of genes encoding functions associated with virulence (Fuchs *et al.* 2010b). Unfortunately, two previous studies have shown that although the *G. mellonella* provides an effective model of *C. albicans* infection**, it is not a useful model for *C. glabrata* disease (Cotter, Doyle and Kavanagh 2000; Bergin, Brennan and Kavanagh 2003). Cotter Doyle and Kavanagh (2000) showed that inoculating *G. mellonella* with 2 × 10^6^ cells of *C. glabrata* NCPF 4733 had no impact on survival at 30°C. While Bergin *et al.* showed that an inoculum of 1 × 10^6^ *C. glabrata* NCPF 4733 resulted in >80% survival of *G. mellonella* larvae at 30°C. Analysis of the *C. glabrata* infected *G. mellonella* showed a reduced fungal burden compared to *C. albicans* with little change in the haemocyte density (Bergin, Brennan and Kavanagh 2003). In contrast, Bates *et al.* have preliminary data that indicate that an inoculum of 5 × 10^5^–1 × 10^6^ cells of *C. glabrata* ATCC 2001 was sufficient to observe approximately 80% mortality in *G. mellonella* 24 h post-infection at 37°C and that it follows a clear dose response, similar to that seen in a mouse model of dose-dependent killing (Steven Bates, Exeter University, pers. Comm.). Thus, *G. mellonella* could develop as an important host for examining *C. glabrata* virulence as at a physiologically relevant temperature of 37°C it is susceptible to *C. glabrata* infection, but not at 30°C. This raises the intriguing possibility that a subset of genes important for *C. glabrata* virulence are switched on at 37°C.

*Drosophila melanogaster* has been used as a work horse of classical genetics for well over a century, and in particular following the discovery of the role of Toll in induction of antifungal responses, its innate immune system has been well studied. Recently, it has been adapted for use as a model to analyse microbial virulence (Lemaitre *et al.* 1996; Alarco *et al.* 2004; Chamilos *et al.* 2006; Panayidou, Ioannidou and Apidianakis 2014; Brunke *et al.* 2015). However, controversy remains over whether wild-type *D. melanogaster* is susceptible to fungal infections, Alarco *et al.* (2004) reported that on average 85% of their wild-type *D. melanogaster* survived when injected with approximately 1 × 10^3^ *C. albicans* cells per fly leading them to conclude that wild-type *D. melanogaster* is highly tolerant to fungal infection. In contrast, Glittenberg *et al.* (2011b) reported that by injecting *C. albicans* cells directly into the *D. melanogaster* haemolymph they observed high mortality systemic infections that were dose dependent; furthermore, their results strongly correlated with previous mouse infection models using the same *C. albicans* strains. Despite the controversy surrounding the use of wild-type *D. melanogaster* as a virulence model for fungal infections, both Toll and Spätzle-deficient *D. melanogaster* are susceptible to fungal infections (Alarco *et al.* 2004; Quintin *et al.* 2013; Panayidou, Ioannidou and Apidianakis 2014).

While the majority of studies investigating the use of *D. melanogaster* as a virulence model have been carried out with *C. albicans*, Quintin *et al.* showed that although wild-type *D. melanogaster* did not succumb to *C. glabrata* infection, they were not able to clear *C. glabrata* even 2 weeks post-infection which is reminiscent of the situation observed in mice where *C. glabrata* can persist for at least 4 weeks without killing immunocompetent mice (Jacobsen *et al.* 2010; Quintin *et al.* 2013). Additionally, they were able to show that *C. glabrata* had triggered the immune system in wild-type *D. melanogaster* by showing a significant increase in the expression of *Drosomycin*, the antifungal peptide gene regularly used as a read-out for Toll-pathway activation following immune challenge (Quintin *et al.* 2013). It is known that *C. glabrata* evades the host immune system by allowing itself to be taken up by macrophages where it continues to proliferate (Seider *et al.* 2011; Brunke and Hube 2013; Brunke *et al.* 2014). Thus, *D. melanogaster* has the potential to be an attractive choice for large-scale screens of mutants as both attenuated strains and strains that are virulent but do not activate the immune response can be identified relatively quickly, especially since *Drosomycin*–GFP-tagged *Drosophila* strains are available allowing *Drosomycin* expression to be observed using a fluorescent microscope (Glittenberg *et al.* 2011a). Furthermore, Toll-deficient *D. melanogaster* do succumb to *C. glabrata* infection and attenuated *C. glabrata* null strains can be identified using Toll-deficient flies; for example Quintin *et al.* observed that Toll-deficient flies injected with a double null *C. glabrata yps1 yps7* (Yps1 and Yps 7 have previously been shown to be required for cell wall integrity and survival inside macrophages) succumbed more slowly compared to flies infected with the wild-type parental strain (Kaur, Ma and Cormack 2007; Quintin *et al.* 2013). Brunke *et al.* have combined the use of Toll-deficient flies and the deletion collection to screen 416 *C. glabrata* mutants to investigate whether results from this model of virulence can be used to predict the outcome of infections in murine models. They showed that virulence in *D. melanogaster* was at least partially predictive of fitness in mice. The model performed much better as an indicator of virulence than *in vitro* growth, which has been used as a proxy for virulence capacity, with slow *in vitro* growth rates taken to indicate a less virulent strain, based on the assumption that the slow growth rate will persist in the host (Brunke *et al.* 2015). Specifically, Brunke *et al.* (2015) observed that although deletion of *SSD1* or *PKH1* had no effect on *in vitro* growth, they were less virulent in a Toll-deficient fly model, while a *cbk2* mutant was strongly deficient in proliferation *in vitro* but was not found to be reduced in virulence in both Toll-deficient flies or a mice model. Thus, Toll-deficient flies may be a useful model to measure the fitness of *C. glabrata* mutants for pre-screening strains for selection for further validation in a mouse model or even for microbial drug target screens, which are currently selected based on *in vivo* growth rates.

However, a significant drawback to using an insect host is that they do not have an adaptive immune system, and it has been documented that, at least in the case of *C. albicans* infections, integration of the innate and adaptive immune system occurs, for instance, phagocytic activity is reinforced by Th1 cytokines and impaired by Th2 cytokines (Romani, Bistoni and Puccetti 1997; Romani 2000). This is corroborated in human studies where acquired immunity to *C. albicans* correlates with Th1 reactivity (La Sala *et al.* 1996; Fidel *et al.* 1997; Romani 2000) while susceptibility to candidosis seen in patients with human immunodeficiency virus infection or in patients with chronic mucocutaneous candidosis correlates with a biased Th2 response (Kobrynski *et al.* 1996; Leigh *et al.* 1998; Romani 2000). To try and obviate this problem and develop a system in which adaptive immunity may be interrogated, Chao *et al.* investigated whether zebrafish (*Danio rerio*) could be used as a virulence model to analyse *C. albicans* infection. Zebrafish were chosen as they have an adaptive component to their immune system, a high reproductive rate, low maintenance costs, have transparent embryos that makes *in vivo* visualization possible and can be manipulated with a comprehensive series of molecular tools (Chao *et al.* 2010). They found that using an inoculum up to 1 × 10^8^ cells resulted in reproducible high mortality systemic infections that were dose dependent and capable of differentiating attenuated strains, for instance 40% viability of the zebrafish was observed 100 h post-injection with *C. albicans hcg1/ hcg1* compared to only 10% viability when injected with the parental wild-type *C. albicans* (Chao *et al.* 2010). In addition, they showed that *C. albicans* is able to colonize, proliferate and invade deep into the organs of zebrafish (Chao *et al.* 2010). The power behind this virulence model can be seen by the publication of a host–pathogen interaction network for a *C. albicans–*zebrafish infection model, which identified several important proteins related to *C. albicans* infection that may prove useful drug targets. At the same time, the study identified important immune defensive mechanisms activated in zebrafish in response to *C. albicans* invasion (Kuo *et al.* 2013; Gratacap and Wheeler 2014). An additional advantage of the zebrafish model is that many transgenic lines contain immune cells constitutively labelled with fluorescent reporters and as an embryo it is optically transparent allowing real-time visualization of pathogen–host interactions. Brothers, Newman and Wheeler (2011) exploited this to visualize the cellular impact of loss of host phagocyte NAPDH oxidase activity in a zebrafish infection model of disseminated candidosis using *C. albicans* and found that *in vivo* the phagocyte NADPH oxidase regulates filamentation of *C. albicans* and that phagocytosis can result in a scenario where *C. albicans* survives and divides but is unable to germinate or lyse the host cell. A drawback to using zebrafish is that they are ectothermic and prefer temperatures of 28°C, while humans are endothermic and have a normal body temperature of 36.8 ± 0.4°C (Chao *et al.* 2010; Brothers, Newman and Wheeler 2011; Gratacap and Wheeler 2014). However, in the case of *C. albicans*, although filamentous growth is enhanced at higher temperatures, fungal germination has been observed *in vivo* at 28°C and many studies have implicated a role of filamentation in virulence at this temperature (Brennan *et al.* 2002; Chamilos *et al.* 2006; Fuchs and Mylonakis 2006; Brothers, Newman and Wheeler 2011). Thus, zebrafish may prove to be a tractable virulence model for real-time visualization of pathogen–host interactions, and although researchers have yet to investigate *C. glabrata* in a zebrafish model, the need to understand pathogen–host interactions in real time makes zebrafish a serious contender for use in future virulence studies. However, caution should be used when investigating *C. glabrata* virulence using a zebrafish model as the *Galleria* virulence data suggests that a subset of genes important for *C. glabrata* virulence are switched on at 37°C since at a physiologically relevant temperature of 37°C it is susceptible to *C. glabrata* infection, but not at 30°C. Nevertheless, we believe the zebrafish model could still be useful as a tool to assess/ screen *C. glabrata* deletion/ mutant strains migration within the host as well as host–pathogen interactions before examining strains further in a mouse model.

Thus, it can be seen that alternative hosts for virulence studies are emerging and their relative simplicity could be used in global screens facilitating analysis of *C. glabrata* infections

### RNA-Seq to reannotate the genome and understand regulation of expression in *C. glabrata*

Because of the recent advances made in next-generation sequencing, such as RNA-seq, which shows a snapshot of the quantity and nature of mRNA expressed in a genome at a given moment in time, the gene prediction models for many species have been corrected and reannotated aiding basic research into these species. Indeed, recently Aoyama *et al.* performed Cap Analysis Gene Expression (CAGE analysis), a method that was introduced to determine the transcription start sites on a genome-wide scale by isolating and sequencing fragments originating from the 5^′^ end of RNA transcripts, on RNA isolated from *C. glabrata* grown in seven different conditions. This analysis suggested the existence of more than 50 previously unannotated *C. glabrata* genes as well as confirming the identity of 4316 genes previously listed on the Candida Genome Database (Aoyama *et al.* 2014). This is in line with the Linde *et al.* (2015) recent work looking at the transcriptional landscape of *C. glabrata* in nutrient-rich media, following nitrosative stress and during pH shift using RNA-seq, which identified 49 novel *C. glabrata* genes. These workers went on to verify four of the novel genes, *C. glabrata* NP4, 11, 32 and 38 via RT-PCR, and showed that their expression was upregulated during a pH shift from pH4 to pH8 suggesting that the encoded proteins play a role in helping *C. glabrata* regulate and/or adapt to changes in environmental pH (Linde *et al.* 2015). *Candida glabrata NP4* and *NP38* were also shown to be downregulated upon interaction with human neutrophils, suggesting that the encoded proteins may be important for *C. glabrata*'s role as a pathogen (Linde *et al.* 2015). Interestingly both Aoyama *et al.* (2014) and Linde *et al.* (2015) found divergence in the transcriptional regulation between *C. glabrata* and *S. cerevisiae*, thereby reinforcing the idea that great care should be taken when extrapolating such regulatory data between species. For example, 252 genes in *S. cerevisiae* have been reported to contain upstream small open reading frames (uORFS) that can inhibit translation of the downstream ORF by interfering with the initiation of translation from its start codon. None of the *C. glabrata* orthologous genes were reported to contain uORFs (Aoyama *et al.* 2014). Instead, based on the CAGE analysis, Aoyama *et al.* predicted 72 *C. glabrata* genes to contain uORFs, yet none of the 72 *S. cerevisiae* orthologues have been reported to harbour uORFs. These data strongly suggest that there is regulatory divergence between these two yeasts (Aoyama *et al.* 2014). Similarly, Linde *et al.* (2015) reported that the transcriptional response of *C. glabrata* to nitrosative stress was different to both *C. albicans* and *S. cerevisiae*, and hypothesized that this difference could be due to *C. glabrata* being able to replicate within macrophages, and thus *C. glabrata* would require a distinct stress response as macrophages exert nitrosative stress on fungal cells after phagocytosis.

In addition, there are many more annotated fungal genomes available today for comparative genomics analysis. Since the publication of the first genome sequence of a free-living eukaryote, *S. cerevisiae*, was published 24 years ago by Goffeau *et al.*, by 2011 a total of 108 fungal genomes had been annotated (Goffeau *et al.* 1996; Haas *et al.* 2011), and the growing list of annotated fungal genomes continues to grow as sequencing technologies improve. With so many fungal genomes readily available, the possibility now exists of performing comparative genomic analysis with *C. glabrata* to predict evolutionary pathways that may have evolved to aid its survival as a pathogen, and to experimentally validate these predictions. For example, Tsai *et al.* (2014) have recently performed comparative genomics on four species of *Taphrina* fungi, a plant pathogen that causes plant deformity, and discovered that *Taphrina* fungi utilized multiple strategies to cope with the host environment that were also found in some yeast species such as aneuploidy of genes. Further advances in next-generation sequencing as well as RNA-seq data in *C. glabrata* will greatly aid our understanding of the unique changes in transcriptional regulation that occur when *C. glabrata* encounters a host and the mechanisms underpinning its virulence.

### New tools that could be adapted for use in *C. glabrata*

Since the introduction of the plasmid to the molecular biology tool kit, technology has advanced greatly and experiments that once took weeks or even years, for example sequencing a genome, can now be done in days or in some cases just a few hours. In this section, the expanding field of genome editing on a global scale will be explored. And although the techniques have not yet been adapted for use in *C. glabrata* research, it is likely that they will be in the very near future.

**Figure 3.**
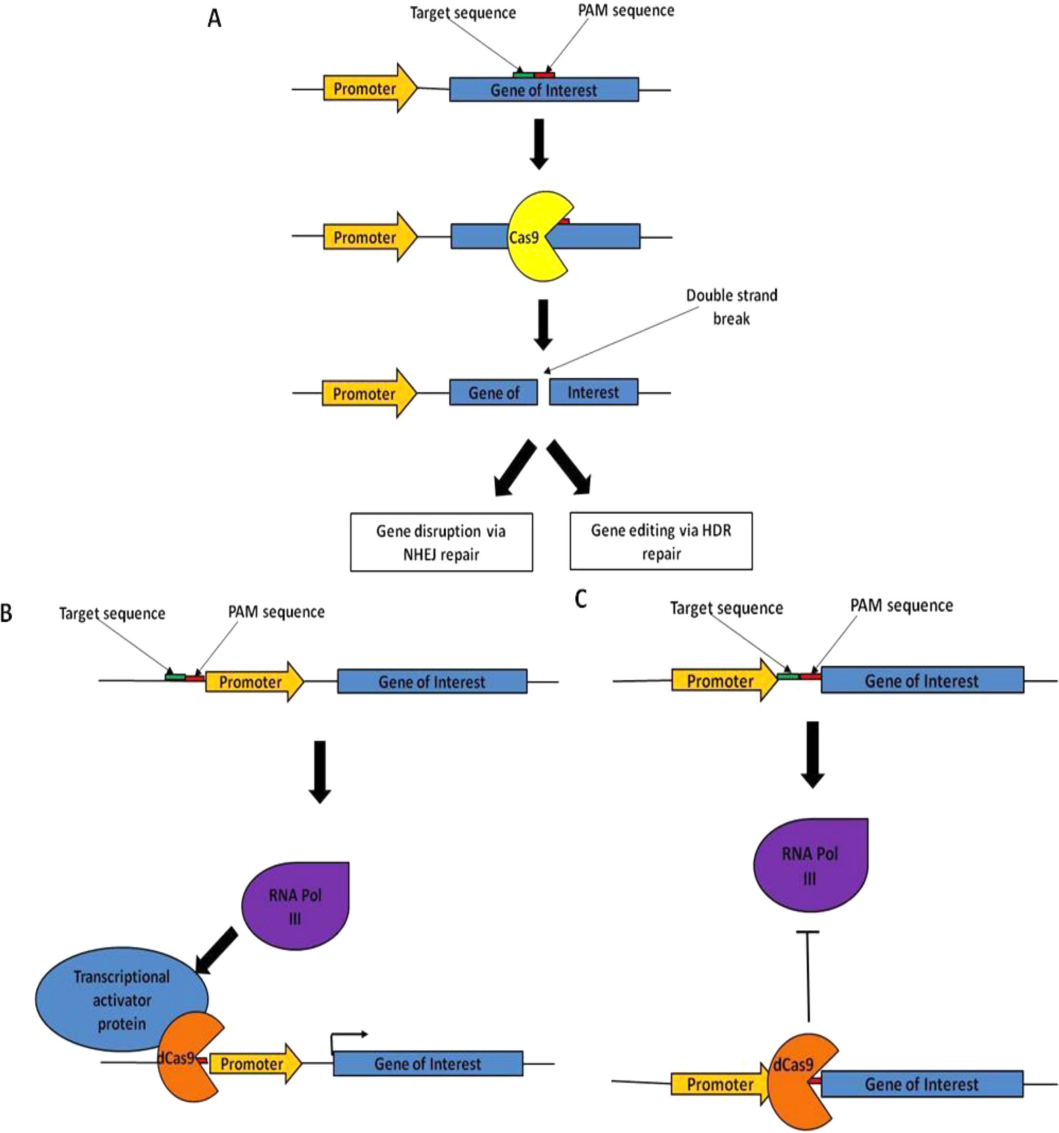
Schematic and applications of the CRISPR-Cas9 system. Archae and bacteria utilize the CRISPR-Cas9 system to degrade foreign DNA by utilizing short RNA strands as guides, and this has been adapted by researchers to (**A**) edit the genome— gRNA strands are synthesized to complement the target sequence and protospacer adjacent motif (PAM) sequence (the PAM sequence must immediately follow the genomic target sequence for Cas9 to bind). The Cas9 enzyme binds to the gRNA and the target sequence and cleaves both strands to form a DSB. The DSB can be repaired via one of two general repair pathways, the NHEJ DNA repair pathway or the homology directed repair (HDR) pathway. Using the CRISPR system, researchers can either disrupt the gene via NHEJ repair (by not providing a suitable repair template) or edit the gene via HDR repair (by transfecting a suitable repair template into the cell at the same time as the gRNA and Cas9). (**B**) Activate gene expression—this utilizes a catalytically inactive form of Cas9 (dCas9) fused to a known transcriptional activator such as VP64. The dCas9–transcriptional activator complex binds to a target sequence just upstream from the promoter and causes upregulation of transcription of the target gene. (**C**) Repress gene expression—by binding dCas9 alone to the target sequence, transcription of the gene is blocked as it prevents the ribosome from binding. Unlike the gene modifications caused by the CRISPR system, both activating and repressing genes using a catalytically inactive form of Cas9 is not permanent as it does not affect the genomic DNA directly.

A technology that has revolutionized and advanced research in the field of artificial gene regulation and genome editing is the Clustered Regularly Interspaced Short Palindromic Repeats (CRISPR)-Cas9 system (Doudna and Charpentier 2014). In nature, CRISPR-Cas systems form part of the adaptive immune system used by archaea and bacteria against foreign DNA. Similar to RNAi, CRISPR-Cas systems utilize short guide RNA (gRNA) strands to direct the degradation of foreign DNA that the system has previously encountered by incorporating fragments of foreign DNA into the CRISPR loci to produce the short gRNAs required to degrade homologous sequences (Mali, Esvelt and Church 2013; Gilles and Averof 2014; Laganà, Shasha and Croce 2014). So far three distinct bacterial CRISPR systems have been identified; Type I, II and III, and it is the Type II system that has mainly been adapted for use in artificial gene regulation and genome editing (Kim and Kim 2014; Shalem, Sanjana and Zhang 2015). The most commonly used CRISPR Type II system consists of a gRNA and an endonuclease, the CRISPR-associated (Cas) nuclease, Cas9, which cleaves the targeted chromosomal DNA in a site-specific manner triggering endogenous DNA repair systems resulting in genome modification (for an in-depth review, see Doudna and Charpentier 2014; Kim and Kim 2014; Shalem, Sanjana and Zhang 2015). Unlike RNAi, the CRISPR-Cas9 system is not limited solely to the silencing or deletion of genes and has been adapted so that it can also be used for editing/modifying the genome via its homology directed repair mechanism, to activate or repress gene expression, to purify regions of genomic DNA and to label genomic DNA for imaging; see Fig. 3 for a schematic and applications of the CRISPR-Cas9 system. For example, Zalatan *et al.* have recently adapted the CRISPR-Cas9 system to generate synthetic multigene transcriptional programs by repressing and activating genes to redirect flux through the violacein biosynthetic pathway (an important metabolic pathway for bacteria) which they recreated in *S. cerevisiae*. Furthermore, their system was designed so that it relied on the expression of the protein dCas9, a nuclease deficient form of Cas9, as a single control point for the activation/deactivation of the multigene transcriptional programs in the system (Zalatan *et al.* 2014). Bao *et al.* (2015) on the other hand have used the CRISPR-Cas9 system to generate multiple gene disruptions in a single step in *S. cerevisiae* representing a powerful tool for creating yeast strains with multiple knockouts in less than a week, and they have termed this the HI-CRISPR system. However, their process will need optimization as they showed that their efficiency varies from disrupting *AFT2, GCY1* and *YR1* in 6 days with a 100% efficiency to an efficiency range as low as 27% in disrupting *CAN1, ADE2* and *LYP1* in 4 days, and this they postulated was due to the efficiency of the DSB introduced by the CRISPR-Cas9 system and the efficiency of the subsequent homologous recombination by *S. cerevisiae* (Bao *et al.* 2015). In addition, Bao *et al.* (2015) found that triple gene disruptions were also affected by the CRISPR-Cas9 array (the genome sequence encoding the gRNA strand that directs the degradation/modification of the gene of interest) used; however, this should be addressable in the near future as rules for choosing efficient CRISPR-Cas9 targeting sites emerge with greater understanding of the CRISPR-Cas9 system. Furthermore, their new multigene disruption HI-CRISPR system utilized a recyclable plasmid, so that the plasmid can be reused to generate further multiple knockouts in the same strain (Bao *et al.* 2015). Potentially, using the HI-CRISPR system a *C. glabrata* strain with six targeted genes knocked out/disrupted could be created in as little as 12 days, whereas it currently takes several weeks to several months to make a double knockout in *C. glabrata*, speeding up research as the construction of knockout/disrupted genes is often a rate-limiting step in advancement.

The use of the CRISPR-Cas9 system has already been successfully developed in both *C. albicans* and *S. pombe* (Jacobs *et al.* 2014; Vyas, Barrasa and Fink 2015) and is believed to represent the future of genome editing in both *C. glabrata* and all other species as it appears to work in most systems tested to date. However, as NHEJ is more dominant over homologous recombination in *C. glabrata* (Ueno *et al.* 2007; Corrigan *et al.* 2013; Cen, Fiori and Dijck 2015) and the CRISPR-Cas9 technology relies on NHEJ for insertions/deletions and homologous recombination for site-specific mutations (Mali, Esvelt and Church 2013; Doudna and Charpentier 2014; Gilles and Averof 2014; Kim and Kim 2014; Sander and Joung 2014), it is probable that using the CRISPR-Cas9 technology to generate site-specific mutations in *C. glabrata* will be highly inefficient due to its preference for NHEJ. Despite this, we believe that site-specific mutations can still be generated in *C. glabrata* using the CRISPR-Cas9 technology by utilizing the *lig4 C. glabrata* strain recently generated by Cen, Fiori and Dijck (2015) as this strain has a disrupted NHEJ pathway and so far appears to be similar to the parental strain on all conditions examined so far. Thus, we believe that CRISPR-Cas9 technology will play a vital role in furthering our understanding of both the biology and virulence of *C. glabrata* as well as many other species.

## CONCLUSION AND OUTLOOK

Since the designation of *C. glabrata* as a pathogen in 1957 by Wickerham (1957), research has focused on investigating its virulence**, the immune response to *C. glabrata* and identifying putative drug targets that can be developed for treatment. However, still today relatively little is known about the molecular basis of virulence. Recent advances in the *C. glabrata* molecular tool box should aid research into its virulence mechanisms, host–pathogen relationship and reveal novel putative drug targets. Thanks to the partial *C. glabrata* deletion collection, high-throughput screening aimed at elucidating novel drug targets can now take place alongside screens for genes involved in virulence by utilizing one of the new virulence models for preliminary screens before moving to the classical mouse model for further corroboration. Additionally, a partial *C. glabrata* ORFeome is being constructed (Ho and Huvet *et al.*, unpublished, Ho *et al.*, unpublished) and once complete will facilitate high-throughput phenotypic screens and basic research. Furthermore, adaptation of CRISPR-Cas9 for use in *C. glabrata* should revolutionize *C. glabrata* research by simplifying its genome editing, for instance, making a knockdown construction of an essential gene in *C. glabrata* will be less time consuming and difficult, enabling more research into essential gene functions in *C. glabrata.* It is likely that a proportion of the essential genes will play an important role in virulence of *C. glabrata*. Furthermore, current research on *C. glabrata* focuses on orthologues of genes involved in virulence in other species yet an entire subset of *C. glabrata*-specific genes remains unexplored. These genes could be extremely important for *C. glabrata*'s success as a pathogen. With the aid of the CRISPR-Cas9 system and novel virulence models, these *C. glabrata*-specific genes could be screened in a high-throughput manner to identify their role in virulence. In short advances in technology and techniques will continue, and with this will come a better understanding of *C. glabrata*'s mechanism of virulence and the promise that novel drug targets will be discovered and targeted by new therapeutics.
